# Improving Compressed Video Using Single Lightweight Model with Temporal Fusion Module †

**DOI:** 10.3390/s23094511

**Published:** 2023-05-05

**Authors:** Tien-Ying Kuo, Yu-Jen Wei, Po-Chyi Su, Chang-Hao Chao

**Affiliations:** 1 Department of Electrical Engineering, National Taipei University of Technology, Taipei City 10608, Taiwan; t106319012@ntut.org.tw (Y.-J.W.);; 2 Department of Computer Science and Information Engineering, National Central University, Taoyuan City 32001, Taiwan; pochyisu@csie.ncu.edu.tw

**Keywords:** deep learning, compression artifacts removal, video coding

## Abstract

Video compression algorithms are commonly used to reduce the number of bits required to represent a video with a high compression ratio. However, this can result in the loss of content details and visual artifacts that affect the overall quality of the video. We propose a learning-based restoration method to address this issue, which can handle varying degrees of compression artifacts with a single model by predicting the difference between the original and compressed video frames to restore video quality. To achieve this, we adopted a recursive neural network model with dilated convolution, which increases the receptive field of the model while keeping the number of parameters low, making it suitable for deployment on a variety of hardware devices. We also designed a temporal fusion module and integrated the color channels into the objective function. This enables the model to analyze temporal correlation and repair chromaticity artifacts. Despite handling color channels, and unlike other methods that have to train a different model for each quantization parameter (QP), the number of parameters in our lightweight model is kept to only about 269 k, requiring only about one-twelfth of the parameters used by other methods. Our model applied to the HEVC test model (HM) improves the compressed video quality by an average of 0.18 dB of BD-PSNR and −5.06% of BD-BR.

## 1. Introduction

As the resolution of video equipment increases, it becomes more important to reduce the recorded video size in order to lessen storage requirements and network transmission burden. Therefore, video compression technology has become more prevalent, with the HEVC [1] video codec gradually becoming the most widely used video standard. HEVC is a block-based video compression standard, and its block splitting style is variable and more versatile in order to effectively handle objects of varying sizes in video frames. Furthermore, HEVC achieves additional compression by predicting between frames, a process known as interframe prediction. Since video compression works by removing some unnecessary and subtle information from the video, when the compression ratio is increased with more information loss, noticeable artifacts appear, affecting perceptional quality significantly. To remove these artifacts, two standard tools are available in the HEVC in-loop filter: the deblocking filter (DBF) [2] and the sample adaptive offset (SAO) [3]. Unfortunately, the improvement of these two tools is limited and the generated results still contain significant artifacts, so there is other work that proposed different ways to improve them.

Taking advantage of the benefits of deep learning techniques, some of the literature developed restoration methods based on it for removing artifacts caused by video compression. These works [4,5,6,7,8] introduced or replaced trained restoration models in the HEVC in-loop filter to improve the compression quality even further. Yang et al. [9] utilized different network architectures to enhance images encoded with different compression types, because interframe prediction artifacts are much more complicated than intraframe prediction artifacts. All of the methods described above are specific to improving HEVC in-loop filter and are limited to a specific codec; they cannot be generalized to other types of codecs.

More general solutions restore the image and video by predicting the original contents based on analyzing the spatial information in a single image with artifacts, which can be easily generalized to other types of codecs, and these solutions are classified as single image restoration methods. Several researchers [10,11,12] designed shallow models to retrieve spatial information and generate restoration results. Unfortunately, the restoration results were unsatisfactory, and thus, a deeper network architecture was adopted [13,14,15,16,17]. Cavigelli et al. [13] adjusted the filter stride in the network to process the feature maps at four different scales to accommodate different object sizes. Zhan et al. [14] proposed a parallel structure with a joint processing of pixel and wavelet domains. Zhang et al. [15] proposed a denoising convolutional network (DnCNN) that used 20 convolutional layers and batch normalization to establish a deeper network. Tai et al. [16] used multiple recursions and designed the memory block inspired by LSTM [18] to avoid the loss of feature information.

More sophisticated methods use more frame information than a single image to restore video frames from artifacts. These multi-frame restoration methods [19,20] will simultaneously input the current frame and adjacent frames, allowing the model to learn to analyze spatial and temporal information and enhance the quality of the current frame using this information. Jia et al. [19] created spatial–temporal residue networks (STResNets) to process the interframe prediction artifacts, which use two convolutional layers with different filter sizes to capture the features. Yang et al. [20] firstly used a support vector machine (SVM)-based detector to determine whether or not the compressed frame is a peak quality frame (PQF), and then QECNN [9] was used to process the current frame if it were a PQF. 

However, there are several issues with the above methods as well as some barriers to resolving this issue using a deep learning-based approach. To predict more accurately in deep learning methods, the number of layers in the neural network is usually increased, as fewer layers is more difficult to handle serious artifacts effectively. This may make the network more difficult to train, and the parameters associated with it will considerably increase, putting additional strain on the operating devices, especially the low-end platform. Another issue is that the artifacts of video compression are complicated due to complex prediction mechanisms, particularly interframe prediction, used in HEVC. Furthermore, because different compression configuration profiles will produce varying degrees of artifacts, it is difficult to develop a capable method to solve complex and diverse compression distortion. We also notice that the models and training methods proposed by some of the other literature work [5,6,7,9,10,11,12,13,14,15,16,19,20] only targeted the luminance channel Y of YCbCr color space. Although the visual impact on the luminance quality is the most significant and dominant, artifacts in the color channels can still have a noticeable impact on the quality of visual perception. Therefore, this work aims to address the abovementioned problems. 

We proposed a single lightweight model based on our previous paper [21] that is capable of managing and restoring quality after various levels of compression. This model was built using a recursive structure and ResBlock [22] to simulate the architecture of DnCNN [15] because the DnCNN has good restoration performance and its architecture is more flexible for improvement. Our model design and training objective functions take color channels into account, allowing the model to solve the problem of chrominance artifacts with minimal complexity increase. In addition, we added dilated convolution [23] and the ConvGRU [24] concept to our model to expand its receptive field and manage temporal features, respectively.

## 2. Proposed Method

**Figure 1:**
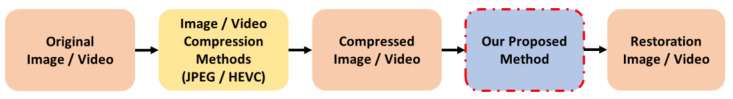
Flowchart of the compression system.

We proposed the model architecture that handles single images and explained how to optimize the model for restoring images and how to solve the problem of color aberration with a low burden of computation. Then, we introduced the setup of the model training, the preprocessing of the datasets, and the design method of the objective function. Finally, we extend our model to video processing to handle the artifacts of interframe prediction, and discuss its architecture and training strategy. Figure 1 is the flowchart of the entire compression system.

### 2.1. Single Image Restoration Method

**Figure 2:**
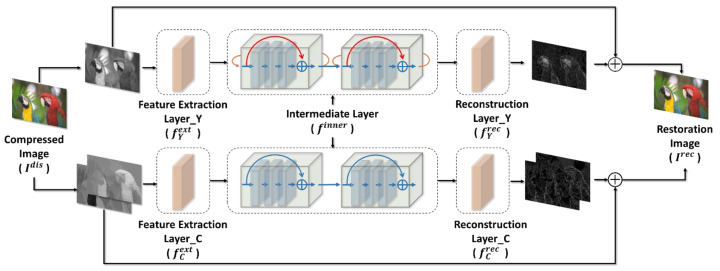
Flowchart of our architecture for a single-image restoration method.

The architecture of deep learning-based restoration methods are commonly made up of three layers: feature extraction, feature processing, and reconstruction. To realize the reconstruction layer, two approaches based on the output of the predicted content can be used: by predicting the whole restored image [6,9,10,11,13,14] or by predicting the residual information for compensating the artifacts [5,7,12,15,16,17,19,20]. Because the amount of information required to predict residual information is less, the residual information prediction is less difficult and the accuracy can be improved. Therefore, our approach adopts the residual-based restoration. We proposed our restoration method based on DnCNN [15]. The architecture of our designed model, shown in Figure 2, predicts the residual image of all YCbCr channels between the compressed and original images. The model can be roughly segmented into three parts: the feature extraction layer, which includes $f_{Y}^{ext}$ and $f_{C}^{ext}$, the intermediate layer $f^{inner}$, and the reconstruction layer, which includes $f_{Y}^{rec}$ and $f_{C}^{rec}$. The operation in the whole model can be expressed as Equations (1) and (2):

(1) $$I_{Y}^{rec}=f_{Y}^{rec}\left(f^{inner}\left(f_{Y}^{ext}\left(I_{Y}^{dis}\right)\right)\right)+I_{Y}^{dis}$$

(2) $$I_{C}^{rec}=f_{C}^{rec}\left(f^{inner}\left(f_{C}^{ext}\left(I_{C}^{dis}\right)\right)\right)+I_{C}^{dis}$$

where $I_{Y}^{dis}\in R^{H\times W\times 1}$ and $I_{C}^{dis}\in R^{H\times W\times 2}$ are the luminance and chroma components of the compressed image, respectively. After the model processing, the reconstructed images $I_{Y}^{rec}\in R^{H\times W\times 1}$ and $I_{C}^{rec}\in R^{H\times W\times 2}$ are obtained. We can then concatenate them back into a three-channel image as Equation (3):

(3) $$I^{rec}=[I_{Y}^{rec},I_{C}^{rec}]$$

Both the feature extraction and the reconstruction layers have a single convolutional layer. We use $f_{Y}^{ext}$ to extract 64-channel features from one luminance channel, while $f_{C}^{ext}$ retrieves the same total number of 64-channel features from both chroma channels as the human visual system is more sensitive to changes in luminance than chroma, and we can use a lighter architecture when designing the chroma model to reduce the complexity and the number of parameters. The feature extraction layers use a filter size of 3 × 3 to extract features. The reconstruction layer generates the residual image for luminance from the 64-channel luminance features and the residual images for the chroma channel from the 64-channel chroma features, which is the inverse concept of the feature extraction layer. That is, only 32-channel features are used to reconstruct each chroma channel. We will explore why we need to process the chroma channel in Section 3.1.

**Figure 3:**
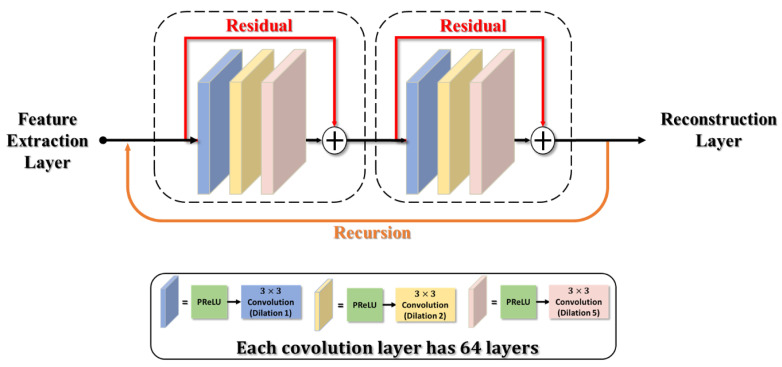
Structure of our intermediate layer.

The intermediate layer contains six convolutional layers as shown in Figure 3. Each convolutional layer uses 64 3 × 3 kernel-sized filters and is activated by PReLU. We use a recursive structure [25] to allow the model to build a deep structure with a small number of parameters. By iteratively performing operations on the previous iteration results, this light structure can achieve a similar effect as in the deep network, and thus deeper features in the image can be extracted that would aid in image restoration. Please note that the recursive structure is only applied to the luminance component.

However, the recursive network could easily cause the problem of gradient vanishing. To address this issue, we integrate our method with the concept of a residual block [22,26] in our intermediate layer. We decompose our six-layer convolutions in our recursive network into two residual block units in a series connection, as shown in Figure 3. It is worth noting that the PReLU location in our design has been swapped with the convolutional layer, which embraces the same concept of the pre-activation design proposed by ResNet-v2 [27] to effectively improve the performance of residual blocks.

If the standard convolution of 3 × 3 kernel size is used in our model, the receptive field would only reach up to 41 × 41. To improve performance with a limited number of layers, we adopt dilated convolution [23,28] in our model to enlarge the receptive field. By adjusting a dilated coefficient to extend the receptive field with the same kernel size, the convolution achieves a larger receptive field. If the dilated coefficient is set to one, the dilated convolution degenerates to the standard convolution. Although the dilated convolution increases the receptive field, the architecture must be set up with extreme caution. Wang et al. [29] pointed out that if the dilated coefficient is not properly set, the gridding issue will appear in the model output. Therefore, we designed our intermediate layer using the hybrid dilated convolution design rules outlined in [29]. For the three convolutional layers, we assigned dilated coefficients of 1, 2, and 5, respectively. The convolution layers in this design can be thought of passing through equivalent filter sizes of 3×3, 5×5, and 11×11.

Our model design is summarized in Table 1. The receptive field of our final model will be increased to 101×101 in the luminance channel. It also improves the receptive field in the chroma channels, increasing it from 16×16 to 37×37. 

To train our model to repair single images, we used two datasets, BSDS500 [30] and DIV2K [31]. These datasets contain uncompressed raw images of various scenes stored in RGB color space. The BSDS500 contains 500 images for training segmentation tasks. DIV2K is a dataset with 900 high-resolution images that has been released for training super-resolution tasks.

We used JPEG compression on the BSDS500 images and a quality factor (QF) of 10 to create compressed images for our initial model training. We used DIV2K to generate an extended dataset by compressing it with various compression quality factors (QF = 10, 20, 30, and 40) to consider different compression qualities. Initially, only the BSDS500 training and test sets were used as the training set for our model, and the remaining BSDS500 images are used as the validation set to fine-tune the model. We used the transfer learning technique when training with the extended datasets to fine-tune the parameters of the model trained on compressed BSDS500 data with a quality factor of 10 in order to reduce training time. The training and testing images were converted to YCbCr color space and cropped into 80 × 80 image patches before feeding the model. When loading data for training, we used three methods of data augmentation to allow the network to learn more diverse characteristics, such as: (1) randomly rotating images by 90°,180°, or 270°; (2) randomly flipping images horizontally; and (3) cropping patches at random locations of images but not around image boundary.

To train the model, a mini-batch stochastic gradient descent method combined with momentum (SGD + m) is used in this work. We started with a learning rate of 0.1 and decreased it to 0.01 and 0.001 at the start of the 100,000th and 200,000th iterations, respectively. Other hyperparameter settings include a batch size of 32 and the use of the He initialization method [32] for parameter initialization.

We used L2 loss as the objective function for model training. In comparison to other methods in the literature that only deal with luminance artifacts, we trained simultaneously in both luminance and chroma channels to achieve better results. As a result, in the objective function, we must account for both the luminance and the chroma. The λ is set to 0.25 in accordance with the 4:1:1 image compression ratio commonly used in the YCbCr field.

(4) $$Loss_{total}=Loss_{Y}+\lambda Loss_{C}$$

### 2.2. Multi-Frame Restoration Method

**Figure 4:**
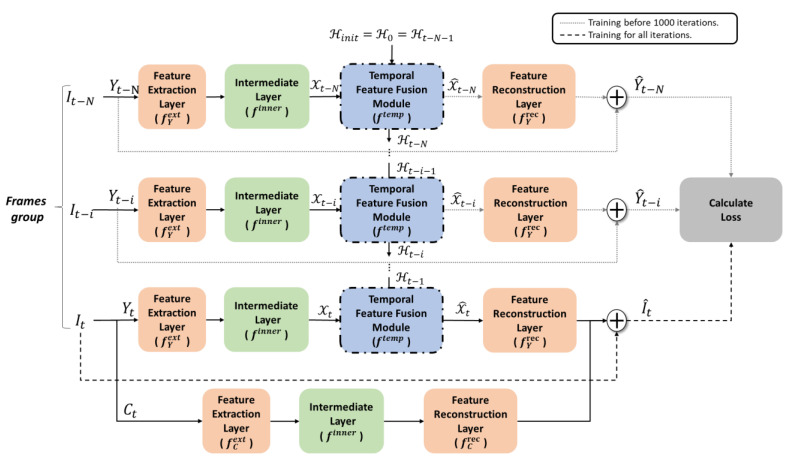
Flowchart of our architecture for multi-frame restoration method.

The video compression algorithm will take the previous frames as the reference, search similar information from the reference, and then apply them to the current frame through motion compensation, resulting in a compressed image with complex compression artifacts from prediction loss. To enable our method to handle the video compression artifacts, we design a temporal feature fusion module $f^{temp}$ that allows us to extend our single-image restoration method to multi-frame restoration method. Figure 4 depicts the architecture of our multi-frame restoration method, in which we utilized $f^{temp}$ to integrate the spatial feature X and temporal feature H from the contiguous frames in the luminance component to retain temporal features of previous frames. In terms of chroma, due to the computational complexity and the fact that chroma is less important than luminance, we continue to use a single image architecture to remove chroma artifacts, and spatial information is sufficient to eliminate visual chroma artifacts. In Figure 4, since the intermediate layer is already able to handle the spatial features of a single frame as discussed previously, we directly use its feature as a spatial feature of the frame $I_{t-i}$ as $X_{t-i}$. Then, we concatenate $X_{t-i}$ with the previous temporal feature $H_{t-i-1}$ to obtain the module input $F_{t-i}=\left(X_{t-i}^{0},X_{t-i}^{1},\ldots ,X_{t-i}^{63},H_{t-i-1}^{0},H_{t-i-1}^{1},\ldots ,H_{t-i-1}^{63}\right),$ where the superscript of X and H indicates the 64-channel features as used in the single-frame restoration scheme, and thus, $F_{t-i}\in R^{H\times W\times 128}$.

**Figure 5:**
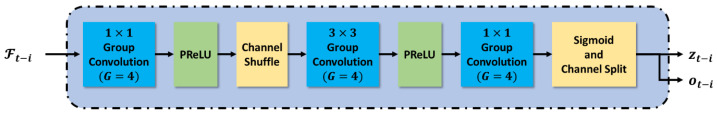
The structure of temporal feature fusion module.

The detailed structure of temporal feature fusion module $f^{temp}$ is shown in Figure 5. We designed the $f^{temp}$ based on the ConvGRU [24,33] concept, and its structure is referred to the ShuffleNet [34] concept to avoid the computational complexity and parameter increase caused by a large number of channels. As shown in Figure 5, the 128 channel features of $F_{t-i}$ are first evenly divided into four groups of channel features, and each group is processed by a group convolutional layer with a size of 1 × 1, and then shuffled via ShuffleNet to process different channels of feature information. Next, it is processed sequentially by the group convolutional layers with sizes of 3 × 3 and 1 × 1. That is, we used three group convolutional layers in total to fuse the $X_{t-i}$ and $H_{t-i-1}$. Finally, the sigmoid function is applied to the output to clip it to [0, 1] as the probability of importance, and the output is divided into two gates, each with 64 channels: the update gate *z* and the output gate *o*. After the features acquired in time and space are fused,$X_{t-i}$ and $H_{t-i-1}$ can be reconstructed and updated using $z_{t-i}$ and $o_{t-i}$ by Equations (5) and (6), respectively, where ∘ denoted the Hadamard product. The residual information for restoring the compressed frame can be generated by a feature reconstruction layer after the new feature ${\hat{X}}_{t-i}$ is calculated, and the hidden state $H_{t-i}$ is passed on to the next frame for processing and updating, as shown in Figure 4.

(5) $${\hat{X}}_{t-i}=o_{t-i}\circ X_{t-i}+\left(1-o_{t-i}\right)\circ H_{t-i-1}$$

(6) $$H_{t-i}=z_{t-i}\circ X_{t-i}+\left(1-z_{t-i}\right)\circ H_{t-i-1}$$

The dataset used to train our model was obtained from Xiph.org [35] in video raw file format. This dataset contains a variety of video data, yet we exclude from training the videos, including animation and above 4K resolution. We used HEVC reference software (HM-16.15) [1] to create the compressed images on Xiph.org, and the compression mode is configured using the HEVC standard low-delay P (LDP) with quantization parameter (QP) values of 22, 27, 32, and 37. Because the compression content in LDP mode includes various prediction methods such as interframe and intraframe prediction, this model is suitable for creating a dataset to train the multi-frame restoration model. To be able to compare with the artifact removal technology in the HEVC standard, we will turn off DBF and SAO to compress the video.

We sample multiple sets of frames in each sequence, and each sample set consists of N+1 consecutive frames to fit the model we designed. In addition, the video content for some video sequences in the dataset is similar for all frames and has countless duplicate information. When we create the dataset for the model training, we will extract only four sets of frames from each video sequence and skip the frame without substantial content. Considering that HEVC LDP configuration uses four frames as a group of picture (GoP), we also use a similar concept to train the model by a set with four frames (N = 3).

In training, we used the same preprocessing as the trained single-image restoration method. The same random rotation, random horizontal flipping, and random cropping are used to augment the number of training samples in all four continuous frames. Because the parameters of our architecture in spatial processing has already been completed in a previous training, we can use transfer learning to fine-tune this pre-trained model, saving us the time of training the video model. The previously trained model aims to improve JPEG compression artifacts so that its spatial features differ slightly from HEVC. Therefore, the parameters of the spatial model have to be gradually updated and adjusted during training, to best solve the HEVC video compression problem.

There is no need to adjust parameters significantly during the training because the pre-trained model has the ability to remove compressed artifacts, so that we set the initial learning rate to 0.01. After the 50,000th and 100,000th iterations, the learning rate was adjusted to 0.001 and 0.0001, respectively, and the model training was completed after updating all model parameters 150,000 times. We use Equation (7) as the objective function for the first 1000 iterations of training, which requires reconstructing N+1 frames in a frame group as shown in Figure 4. To speed up the training, after 1000 iterations, the objective function in Equation (4) is applied to the model, which only measures for the current frame instead of N+1 frames to save the complexity. λ is set as 0.25 in our objective functions.

(7) $$Loss=\sum_{i=0}^{N}Loss_{Y_{t-i}}+\lambda Loss_{C_{t}}$$

## 3. Experiment Results

In our experiment, model training and testing were performed on a personal computer equipped with an Intel i7-4790 CPU, an NVIDIA 1080 8 GB GPU, and the Ubuntu operating system. We used Python and the deep learning framework PyTorch for our neural network model implementation, as well as the HEVC reference software HM16.15 for video compression. To assess the restoration methods, we used the PSNR, SSIM [36], and PSNR-B [37] with larger values indicating higher quality. The PSNR is the most straightforward and widely used method for determining the difference between the reference and evaluated images. In related studies, additional quality evaluation methods, SSIM, and PSNR-B were used to make the image quality assessment more comparable to the human vision. SSIM is currently the most prevalent evaluation method based on human visual system characteristics. There is usually a strong correlation between adjacent pixels in natural images, and the human eye is sensitive to differences in local information. Therefore, unlike PSNR, which computes point-to-point differences in pixels, SSIM computes through the comparison of image blocks. PSNR-B is a method for evaluating block-based compressed images that uses PSNR with the blocking effect factor (BEF) added. BEF calculates the discontinuity of the horizontal or vertical boundary pixels of the blocks in the image to determine the degree of blocking artifacts.

### 3.1. Experiment of Single-Image Restoration Method

Table 2 summarized the dataset used in the experiment of single image compression artifacts. The training of datasets BSDS500 and DIV2K has been described in Section 2.1. We test our proposed single image retorsion methods using JPEG compression on the LIVE1 [38,39] dataset. We applied the same quality factor (QF) to 10, 20, 30, and 40 as we had to the training set.

We conducted ablation tests to validate our model design by gradually adding each model component to the model and then analyzing the gains and efficiency of each added model component. The basic model consists of eight convolutional layers with a 3×3 kernel size. The test model components include recursive networks, residual blocks, and dilated convolution that we introduced in Section 2.1. Finally, we will go over model training after chroma has been added. Table 3 shows the evaluation results of the trained models. The images tested here were compressed using a JPEG quality factor of 10. According to the data tested by each model, our model gradually increases in performance as the design is optimized. After adding the color processing, there is only a slight decrease in Y channel quality, but there is a significant increase in chroma channels. In the end, the performance of our proposed network is further improved by training with DIV2K images, allowing it to deal with all other QFs using the same model parameters.

**Figure 6:**
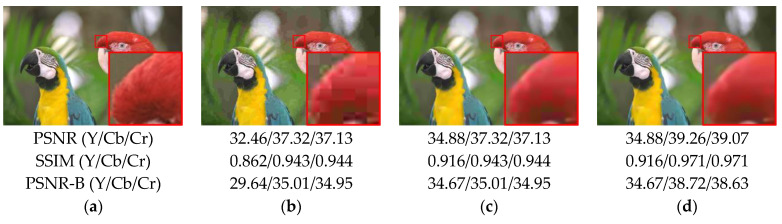
Comparison of compressed and restored images on parrots from LIVE1 (QF = 10). (**a**) Ground truth; (**b**) compressed image; (**c**) image with only Y channel restored; (**d**) image with both Y and CbCr channels restored.

**Figure 7:**
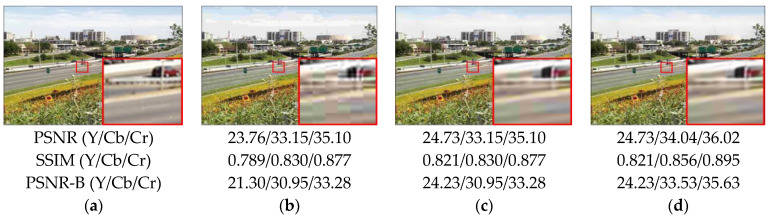
Comparison of compressed and restored images on Flowersonil35 from LIVE1 (QF = 10). (**a**) Ground truth; (**b**) compressed image; (**c**) image with only Y channel restored; (**d**) image with both Y and CbCr channels restored.

Figure 6 and Figure 7 show how the addition of a color processing component improves not only the evaluation metrics, but also the visual quality. In Figure 6b,c, if our proposed method only deals with luminance, the overall artifacts of the image have already been improved greatly when compared to original compressed images, but there are still visible color artifacts. Figure 6d shows how the color block that occurred in Figure 6c can be effectively restored. We can obtain the same improvement results by referring to Figure 7. It proves the importance of restoring color artifacts, which is why we developed our method for processing both the luminance and chroma.

Table 4 compares the results of our method to those of other studies [10,12,13,14,15,16,17] tested in the LIVE1 dataset. Since no other existing work provides data for the chroma channel, we will only compare our chroma improvement to the chroma quality of JPEG-compressed images. Moreover, since the SSIM settings used in the studies conducted in [14,15,16] are not the same as those in other works, we only use PSNR and PSNR-B for comparison. The values in parentheses represent the percentage improvement of our proposed method compared to other methods. 

Among all the methods, only our method can manage the different degree of artifacts using the same model parameters, and the results evaluated under both PSNR and PSNR-B are very close to the first place, with the difference not exceeding 0.1 dB at most. It is worth noting that the parameter amount of these methods ranges from 3 to 22 times that of our model, and several sets of model parameters must be prepared for various degrees of artifacts, so the total parameter amount is several times higher than that indicated in Table 4.

### 3.2. Experiment of Multi-Frame Restoration Method

To create the test set for evaluating the multi-frame restoration model performance, we employed the same settings as the training set to compress the video from HEVC test sequences [40]. We used the low-delay profile (LDP) in the HEVC default configuration, with QP values 22, 27, 32, and 37, and disabled the in-loop filter. We do not test Class F, containing artificially produced sequences in the HEVC test sequences, because our model was developed for natural scenes. The evaluation can fairly justify whether our method can effectively remove the artifacts by utilizing the relationship between the frames. Table 5 summarizes all of the datasets used to train and test our multi-frame restoration method. 

**Figure 8:**
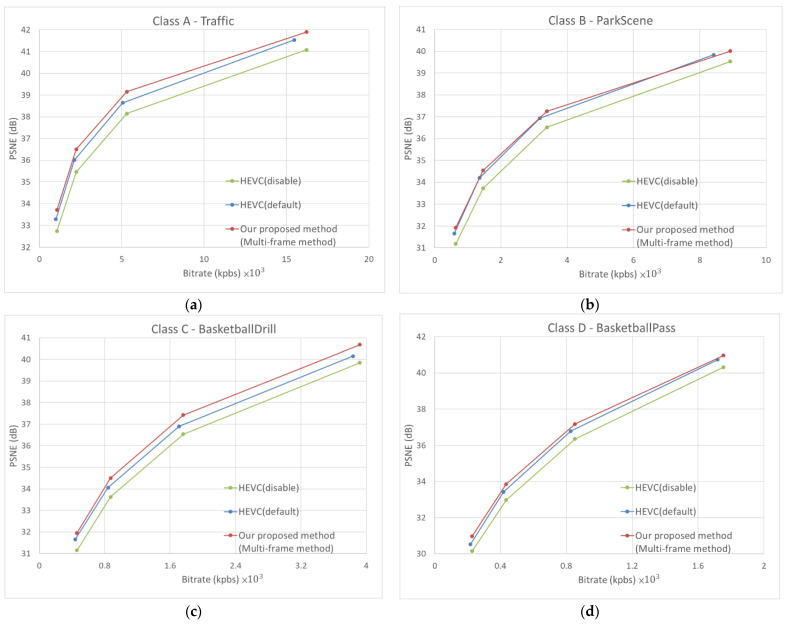

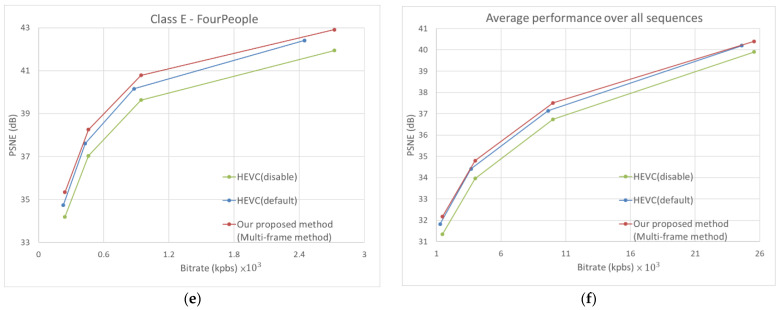
RD-curve results for difference sequence using QP = 22, 27, 32, and 37. (**a**) Class A—Traffic; (**b**) Class B—ParkScene; (**c**) Class C—BasketballDrill; (**d**) Class D—BasketballPass; (**e**) Class E—FourPeople; (**f**) average performance over all sequences.

We used the BD-PSNR and BD-BR proposed by Bjontegaard et al. [41] to compare and measure the visual quality difference and bitrate difference, respectively, so as to evaluate the performance of video restoration. The higher the BD-PSNR is, the better the encoded quality is, while the lower the BD-BR is, the fewer bitrates are required for the encoded bitstream. As shown in Table 6, our proposed method can manage artifacts caused by HEVC compression with disabled DBF and SAO. In comparison to HEVC encoding with enabled SAO and DBF, our method improves BD-PSNR by 0.18 dB, while reducing BD-rate by 5.06%. Figure 8 presents the rate-distortion (RD) results obtained for randomly selected sequences in each class (Traffic from Class A, ParkScene from Class B, BasketballDrill from Class C, BasketballPass from Class D, FourPeople from Class E), as well as the overall performance. These figures represent the PSNR performance comparison between our proposed method and HEVC on luminance frames. It can be seen that the PSNR gain of our method is higher than the default mode of HEVC for different classes of sequences and QP values. 

**Figure 9:**
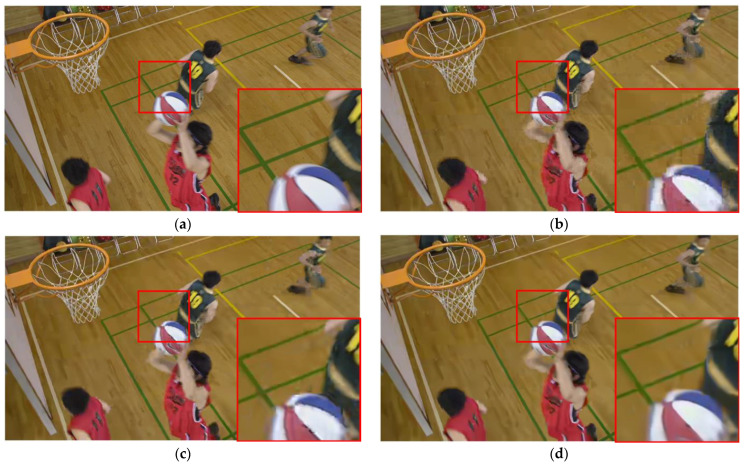
Comparison of visual experience in BasketballDrill from HEVC test sequences (QP = 37). (**a**) Ground truth; (**b**) HEVC disabled in-loop filter; (**c**) HEVC baseline; (**d**) our proposed method.

**Figure 10:**
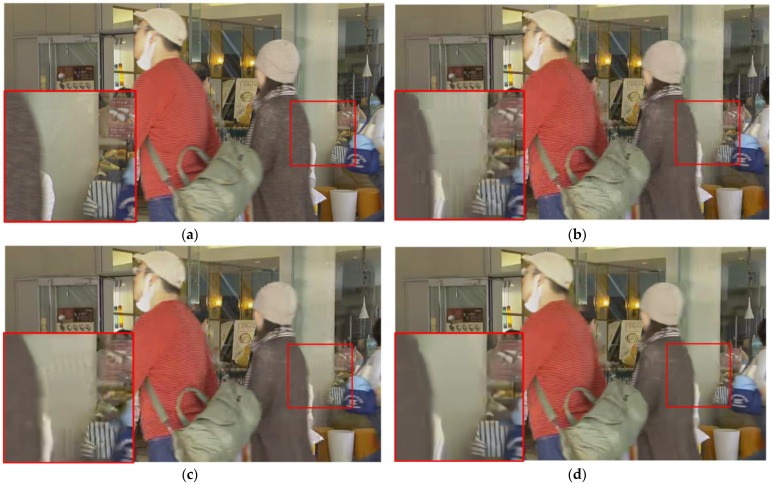
Comparison of visual experience in BQMall from HEVC test sequences (QP = 37). (**a**) Ground truth; (**b**) HEVC disabled in-loop filter; (**c**) HEVC baseline; (**d**) our proposed method.

**Figure 11:**
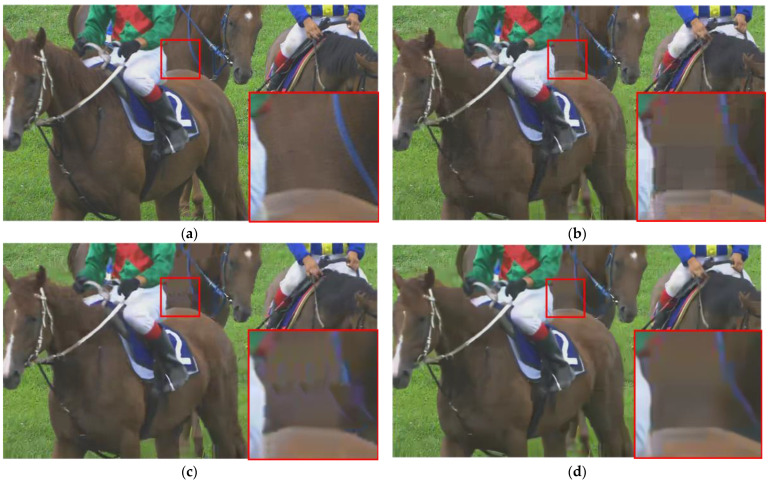
Comparison of visual experience in RaceHorses from HEVC test sequences (QP = 37). (**a**) Ground truth; (**b**) HEVC disabled in-loop filter; (**c**) HEVC baseline; (**d**) our proposed method.

Figure 9, Figure 10 and Figure 11 present a visual comparison of the HEVC disabled in-loop filter, the HEVC baseline, and our proposed method. It is clear that our method can effectively remove not only blocking and ringing artifacts, but also temporal artifacts such as edge floating [42].

Although the blocking artifacts visible in the HEVC disabled in-loop filter are absent in the HEVC baseline results, temporal artifacts and color blocks are present. For example, there is a grain artifact in the ball in Figure 9c, a jerkiness artifact in the wall in Figure 10c, and mosquito noise in the horse in Figure 11c. Our method effectively removes all of the above types of artifacts caused by video compression, as shown in Figure 9d, Figure 10d, and Figure 11d.

We compare our proposed model to other multi-frame restoration methods [5,6] that operate in LDP configuration. The data provided in these works are used to compare the model restoration ability. Because RHCNN [5] does not use the dataset in the same way that our method and He et al. [6] do, we compare our restoration performance to RHCNN [5] separately in Table 7.

In comparison to RHCNN [5], the authors used the majority of the HEVC test sequences as their training set, and the test sets are mostly the same as our Xiph [35] training sets. The comparison in Table 7 is for sequences that both belong to the same test set, and this table compares the I frame and P frame separately by referring to RHCNN [5] comparison fashion. The results show our model significantly outperforms RHCNN in almost all cases, with the exception of the I frame type in the PeopleOnStreet sequence.

Next, we compare our method to that of He et al. [6]. He et al. [6] use postprocessing to improving the quality of the standard HEVC, enabling both SAO and DBF. Our model can not only completely take on varying degrees of compression artifacts, as previously shown, but our model also approaches the performance of the model trained for a specific QP value in [6], as shown in Table 8. Please keep in mind that in Table 8, we compare PSNR rather than BD-PSNR to match the presentation in [6]. Comparing PSNR in a fixed QP is not very informative because the real bitrate usage differs between the two methods even when the same QP is used. Table 8 simply indicates that both methods are of the same quality, even if their performance is comparable.

Table 6, Table 7 and Table 8 shows that our model has nearly the same restoration capability as other methods [5,6]. Although our method may slightly lose the score in some cases, we only need to use one model to manage all cases of QPs, unlike other methods [5,6] that train a specific model for each QP. As shown in Table 9, our model is much lighter than other methods, requiring only about one-twelfth of the parameters (269k vs. 3098k/3340k) used by other methods. Furthermore, if we are to manage four QPs, the amount of our parameters will only be about 1/48 of theirs.

## 4. Conclusions

We have developed a lightweight method for removing compression artifacts. To achieve this, we employed a recursive network that employs only eight layers of convolutional parameters to simulate a 20-layer CNN and adopted dilated convolutions to increase the receptive field without increasing the depth of the networks. To remove the artifacts caused by video compression, we designed a module that fuses temporal features to extract the features between the luminance components of adjacent frames, and then established a single model using a single set of parameters for all levels of compression qualities and predicted frame modes. In comparison to previous CNN-based compression artifact removal work, our method can effectively remove both luminance and chroma artifacts to improve the overall visual quality. In addition, our model integrates spatial and temporal information to reduce artifacts caused by inter-prediction in video. Our method only requires 1/48 of the parameter amount of these methods to achieve the comparable performance. 

## Figures and Tables

**Table 1:** Summary of the filter settings of each layer in our model.

Layer			Filter Size	Dilation Rate	No. of Input Channel	No. of Output Channel	Recursion	Receptive Field
Feature extraction		Y	3×3	1	1	64	No	3×3
		C			2			
Intermediate	Residual block 1	Conv 1	3×3	1	64	64	3 times for Y No for C	97×97 for Y, 33×33 for C
		Conv 2	3×3	2				
		Conv 3	3×3	5				
	Residual block 2	Conv 1	3×3	1				
		Conv 2	3×3	2				
		Conv 3	3×3	5				
Reconstruction		Y	3×3	1	64	1	No	3×3
		C				2		
Total		Y						101×101
		C						37×37

**Table 2:** Summary of image datasets.

Name	No. of Images	Resolution	Usage
BSDS500 [30]	500	481 × 321 or 321 × 481	Training
DIV2K [31]	900	A minimum of 2k pixels along one dimension	Fine-tune pre-trained model by BSDS500
LIVE1 [38,39]	29	480 × 720 to 512 × 768	Testing

**Table 3:** Average PSNR for the LIVE1 dataset (QF = 10).

	PSNR (dB)	SSIM	PSNR-B (dB)
	Y/Cb/Cr	Y/Cb/Cr	Y/Cb/Cr
JPEG	27.77/36.43/36.78	0.791/0.905/0.915	25.33/34.43/34.89
Basic model	29.10/−/−	0.826/−/−	28.77/−/−
+ recursive	29.17/−/−	0.827/−/−	28.80/−/−
+ residual blocks	29.27/−/−	0.829/−/−	28.94/−/−
+ dilated convolution	29.28/−/−	0.829/−/−	28.96/−/−
+ color processing	29.27/38.34/38.58	0.829/0.937/0.942	28.94/38.27/38.52
Our proposed method (fine-tune on DIV2K)	29.35/38.36/38.56	0.831/0.938/.0943	29.01/38.17/38.35

**Table 4:** Comparison of performance to other works on the LIVE1 dataset.

QF	Metrics (dB)	JPEG	ARCNN [10]	L8 [12]	DnCNN [15]	CAS-CNN [13]	MemNet [16]	Zhan et al. [14]	ASQE-CNN [17]	Our Proposed Method		
										Y	ΔCb	ΔCr
10	PSNR	27.77 (5.69%)	28.98 (1.28%)	29.08 (0.93%)	29.2 (0.51%)	29.44 (−0.31%)	29.45 (−0.34%)	29.38 (−0.1%)	29.42 (−0.24%)	29.35	1.59	1.77
	PSNR-B	25.36 (14.24%)	28.7 (0.94%)	28.71 (0.91%)	28.96 (0.03%)	29.19 (−0.75%)	-	29.08 (−0.38%)	-	28.97	3.27	3.45
20	PSNR	30.07 (5.55%	31.29 (1.44%)	31.51 (0.73%)	31.59 (0.47%)	31.7 (0.13%)	31.83 (−0.28%)	31.78 (−0.13%)	31.79 (−0.16%)	31.74	1.26	1.64
	PSNR-B	27.61 (12.93%)	30.76 (1.37%)	30.92 (0.84%)	31.16 (0.06%)	30.88 (0.97%)	-	31.25 (−0.22%)	-	31.18	3.50	3.85
30	PSNR	31.41 (5.51%)	32.69 (1.38%)	-	32.99 (0.45%)	-	-	33.18 (−0.12%)	-	33.14	1.01	1.37
	PSNR-B	28.97 (11.94%)	32.15 (0.87%)	-	32.45 (−0.06%)	-	-	32.54 (−0.34%)	-	32.43	3.47	3.79
40	PSNR	32.36 (5.38%)	33.63 (1.4%)	-	33.96 (0.41%)	34.1 (0%)	-	34.15 (−0.15%)	34.16 (−0.18%)	34.10	0.83	1.22
	PSNR-B	30.02 (11.13%)	33.12 (0.72%)	-	33.43 (−0.21%)	33.68 (−0.95%)	-	33.52 (−0.48%)	-	33.36	3.42	3.78
No. of parameters		-	106k	290k	558k	5145k	667k	961k	890k	224k		
For QFs No.		-	1	2	1	1	1	1	1	4		

**Table 5:** Summary of video datasets.

Name	No. of Sequences	Resolution	Usage
Xiph.org [35]	62	176 × 144, 352 × 288,352 × 240, 704 × 576, 1280 × 720, 1920 × 1680,	Training
HEVC test sequences [40]	20	416 × 240, 832 × 480, 1280 × 720, 1920 × 1680, 2560 × 1600	Class A–E for testing

**Table 6:** BD-PSNR and BD-BR comparison to the HEVC baseline with LDP configuration.

Class	Sequences	BD-PSNR (ΔdB)			BD-BR(Δ%)		
		Y	U	V	Y	U	V
A	Traffic	0.32	−0.02	0.03	−10.04	1.38	−1.46
	PeopleOnStreet	0.26	0.05	0.16	−5.58	−1.84	−8.35
	Nebuta	0.04	−0.06	−0.08	−0.13	3.45	6.22
	SteamLocoMotive	−0.02	−0.03	−0.04	2.34	5.66	9.72
B	Kimono	0.00	0.03	−0.07	0.08	−1.23	4.93
	ParkScene	0.09	−0.02	−0.09	−2.95	1.37	6.38
	Cactus	0.11	0.02	0.01	−4.49	−1.80	0.27
	BQTerrace	0.06	−0.06	0.12	−5.68	6.88	−15.55
C	BasketballDrive	0.06	0.07	0.10	−2.22	−4.40	−4.37
	RaceHorses	0.02	0.07	0.17	−0.57	−3.14	−7.71
	BQMall	0.20	0.14	0.25	−5.20	−6.09	−9.54
	PartyScene	0.16	0.04	0.12	−4.09	−1.88	−4.75
	BasketballDrill	0.34	0.02	−0.25	−8.20	−0.64	8.36
D	RaceHorses	0.26	0.17	0.27	−5.42	−6.23	−9.41
	BQSquare	0.26	0.09	0.21	−7.66	−6.69	−13.93
	BlowingBubbles	0.26	0.09	0.16	−6.41	−3.72	−6.04
	BasketballPass	0.23	0.20	0.24	−4.56	−6.41	−7.11
E	FourPeople	0.38	0.08	0.17	−10.91	−3.49	−7.23
	Johnny	0.23	0.02	0.04	−10.93	−1.97	−3.38
	KristenAndSara	0.25	0.06	0.13	−8.52	−2.50	−6.64
Average		0.18	0.05	0.08	−5.06	−1.66	−3.48

**Table 7:** Comparison of our method to RHCNN [5] using HEVC test sequences.

Sequence	Frame Type	RHCNN [5]		Our Proposed Method	
		BD-Rate	BD-PSNR	BD-Rate	BD-PSNR
PeopleOnStreet (2560 × 1600)	I	−6.10%	0.30	−4.59%	0.23
	P	−3.78%	0.15	−5.52%	0.25
Traffic (2560 × 1600)	I	−5.30%	0.27	−6.56%	0.35
	P	−7.58%	0.19	−10.02%	0.31
RaceHorses (416 × 240)	I	−5.60%	0.29	−6.31%	0.46
	P	−6.25%	0.22	−5.38%	0.26

**Table 8:** Comparison of our method to [6] in terms of PSNR (ΔdB) (QP = 37).

Class	Sequence	He et al. [6]	Our Proposed Method
A	Traffic	0.39	0.43
	PeopleOnStreet	0.64	0.55
	SteamLocoMotive	0.22	0.08
B	ParkScene	0.20	0.28
	Cactus	0.34	0.32
	BQTerrace	0.38	0.25
C	BasketballDrive	0.35	0.35
	BQMall	0.36	0.41
	BasketballDrill	0.47	0.31
D	RaceHorses	0.41	0.43
	BlowingBubbles	0.26	0.37
	BasketballPass	0.40	0.44
E	FourPeople	0.62	0.60
	Johnny	0.54	0.54
	KristenAndSara	0.59	0.57
Average		0.41	0.40

**Table 9:** Comparisons of the number of parameters to other methods based on the multi-frame restoration model.

	RHCNN [5]	He et al. [6]	Our Proposed Method
The number of parameters	3340k	3098k	269k
For the number of QPs	1	1	4

## Data Availability

Not applicable.
